# Age and beliefs about vaccines associated with COVID-19 vaccination among US Veterans

**DOI:** 10.1017/ash.2023.446

**Published:** 2023-10-23

**Authors:** Geneva M. Wilson, Cara E. Ray, Ibuola O. Kale, Aaron M. Scherer, Howard S. Gordon, Frances Weaver, Charlesnika T. Evans, Kevin Stroupe

**Affiliations:** 1 Edward Hines, Jr. VA Hospital, Hines, IL, USA; 2 Department of Preventive Medicine, Feinberg School of Medicine, Northwestern University, Chicago, IL, USA; 3 Carver College of Medicine, University of Iowa, Iowa City, IA, USA; 4 Jesse Brown VA Medical Center, Chicago, IL, USA; 5 University of Illinois Chicago, Chicago, IL, USA; 6 Parkinson School of Health Sciences and Public Health, Loyola University Chicago, Maywood, IL, USA

## Abstract

This project surveyed Veterans’ COVID-19 vaccination beliefs and status. 1,080 (30.8%) Veterans responded. Factors associated with being unvaccinated, identified using binomial logistic regression, included negative feelings about vaccines (OR = 3.88, 95%CI = 1.52, 9.90) and logistical difficulties such as finding transportation (OR = 1.95, 95%CI = 1.01, 3.45). This highlights the need for education about and access to vaccination.

## Introduction

The Veterans Health Administration (VHA) has a unique responsibility to protect patients from COVID-19. Previous, non-VA based, studies have shown that being African American,^1^ Hispanic,^2^ and younger^3^ were associated with decreased vaccination. Vaccinated Veterans were more often male, older, and had more comorbidities associated with serious COVID-19 complications than unvaccinated Veterans.^4^ While these previous reports provided important information about demographic characteristics associated with Veterans’ COVID-19 vaccination, attitudes and beliefs regarding COVID-19 vaccination were not explored. This project aimed to assess Veteran’s perceptions and beliefs surrounding COVID-19 vaccination and its association with COVID-19 vaccination status.

## Methods

Two independent cross-sectional surveys (Wave 1, Wave 2) were conducted. These surveys were designated as quality improvement by the Edward Hines, Jr. VA Hospital Institutional Review Board. Wave 1 surveys were mailed and collected between March 4 – May 19, 2021. Wave 2 was mailed and collected between May 15 – August 13, 2021. Survey responses were combined across waves for this analysis as there was no difference between surveys in factors associated with vaccination.

Wave 1 Veterans were identified from VA facilities with ≤4% COVID-19 vaccination rates (from the VHA Support Service Center (VSSC) “COVID Vaccine Surveillance Dashboard”) as of February 1, 2021. Veterans with VA utilization within the previous 2 years, not in long-term care, with valid US mailing addresses, and complete information on race and ethnicity were included. A stratified random sample of 2,500 Veterans was created with 500 Veterans in each of the following racial/ethnic categories: Non-Hispanic White, Non-Hispanic Black, Hispanic, Native American, and all others.

Wave 2 Veterans were identified from VA hospitals with COVID-19 vaccination rates of ≤33% as of April 26, 2021. Inclusion criteria were the same as Wave 1. No Wave 1 Veterans were included in Wave 2. A final sample of 1,000 Veterans stratified into 500 males and 500 females (to ensure adequate representation of females and identify any possible sex differences), with the five race/ethnicity groups from Wave 1 evenly distributed within each sex was created.

The survey included questions on current COVID-19 vaccination status, willingness to receive a two-dose COVID-19 vaccine, perceived safety and effectiveness of available COVID-19 vaccines, general attitudes regarding vaccination, and reasons for or against COVID-19 vaccination. These items were drawn from co-investigators’ previous survey efforts^5,6^ and prior frameworks for assessing vaccination attitudes and intentions.^7,8^ Returned surveys were entered and managed using REDCap (Research Electronic Data Capture). Age, sex, geographic region, and rurality were extracted from the VA Corporate Data Warehouse (CDW) and merged with survey data.

The main outcome was vaccination status. If participants indicated at any point on the survey that they had received the COVID-19 vaccine, they were classified as vaccinated. All others were classified as “not vaccinated.” The association between demographic characteristics and survey responses was evaluated by vaccination status using chi-square/Fischer’s exact tests for categorical variables and t-tests for continuous variables. Multivariable binomial logistic regression was used to assess the independent association between these factors and vaccination status. Variables with a *p*-value less than 0.05 were included in the final model. Data were analyzed using Stata MP software, version 17 (StataCorp, College Station, TX, USA).

## Results

There was no difference between the recruitment facilities and non-recruitment facilities by geographical region, facility complexity, or urban/ rural designation for Wave 1. There were more mid-level and Southern facilities represented among Wave 2 recruitment facilities. 1,080 (30.8%) Veterans responded: 818 in Wave 1; 262 in Wave 2. Respondents were male (80%) and non-Hispanic White (31%). The mean age was 64.2 years (SD = 14.1). Non-respondents were more likely to be under 50 years old (47.2% versus 15.7%, respectively; *p* < .001), female (27.5% versus 19.7%; *p* < .001), and Black non-Hispanic (21.1% versus 17.6%) or Hispanic (21.7% versus 16.3%; *p* < .001). Overall, 65% of Veterans reported receiving a COVID-19 vaccination (Wave 1: 60%, Wave 2: 81%). Unvaccinated respondents were more likely to be female (22.5% vs 18.2% (*p* = 0.094)) and under age 50 (28% vs 9% (*p* < 0.001)) (Table 1).

**Table 1.** Respondent demographics and comorbidities by survey wave and vaccination status (*N* = 1,080)

Variable	Total		
	Unvaccinated (*N* = 378)	Vaccinated (*N* = 702)	*p*-value
Age			**<0.001**
	106 (28.0)	64 (9.1)	
50–74	243 (64.3)	437 (62.3)	
≥75	29 (7.7)	201 (28.6)	
Gender			0.094
Male	293 (77.5)	574 (81.8)	
Female	85 (22.5)	128 (18.2)	
US Geographic Region			0.584
West	108 (28.6)	196 (27.9)	
Northeast	21 (5.6)	32 (4.6)	
Midwest	53 (14.0)	97 (13.8)	
South	195 (51.6)	369 (52.6)	
Alaska/Hawaii	1 (0.3)	8 (1.1)	
Rurality			
Urban	264 (69.8)	504 (71.8)	
Rural	112 (29.6)	192 (27.4)	
Highly Rural	2 (0.5)	6 (0.9)	
Race/ethnicity			0.253
White non-Hispanic	104 (27.5)	230 (32.8)	
Black non-Hispanic	66 (17.4)	121 (17.2)	
Hispanic	90 (23.8)	135 (19.2)	
Other	68 (18.0)	135 (19.2)	
Multiracial	41 (10.9)	60 (8.6)	
Missing	9 (2.4)	21 (3.0)	
Highest level of reported education			0.715
High school or less	135 (36.2)	259 (37.7)	
Associate’s degree	97 (26.0)	169 (24.6)	
Bachelor’s degree	75 (20.1)	136 (19.8)	
Graduate degree	49 (13.1)	100 (14.6)	
Prefer not to Answer	16 (4.3)	19 (2.8)	
Missing	1 (0.3)	4 (0.5)	
Charlson Comorbidity Index (mean, SD)	1.6 (1.9)	1.5 (1.8)	0.134
Care Assessment Need Score (mean, SD)	45.5 (30.1)	48.2 (27.4)	0.579
Respondent Perceived Health Status			0.542
Poor	13 (3.4)	41 (5.8)	
Fair	88 (23.3)	166 (23.7)	
Good	154 (40.7)	287 (40.9)	
Very Good	97 (25.7)	170 (24.2)	
Excellent	22 (5.8)	31 (4.4)	
Missing	4 (1.1)	7 (1.0)	

Respondents mostly believed that the COVID-19 vaccines were effective (62.8%) and safe (76.0%). However, unvaccinated respondents more often believed COVID-19 vaccines were ineffective (21.4% versus 2.3%, *p* < 0.001), unsafe (21.2% versus 0.9%, *p* < 0.001), and were more worried about vaccine side effects (32.0% versus 11.3%, *p* < 0.001) than vaccinated respondents (Supplemental Table 1). Unvaccinated respondents more frequently expressed disagreement about the general safety of vaccines compared to vaccinated respondents (14.6% versus 2.4%, *p*-value < 0.0001).

Unvaccinated respondents reported logistical concerns about receiving a two-dose vaccine more frequently than vaccinated respondents (Supplemental Table 1). Finding time to schedule a second appointment (11.9% vs 3.4%, *p*-value < 0.0001) having to schedule two appointments within 30 days (14.8% vs 5.0% *p*-value < 0.0001) and concerns that a second dose of the vaccine would increase the risk of side effects (27.5% vs 8.7% *p*-value < 0.0001) were the primary logistical concerns identified by unvaccinated Veterans.

In multivariable binomial regression modeling, factors associated with increased odds of being unvaccinated were being under 50 years of age (OR 7.68, 95%CI = 4.36, 13.51), negative (OR = 3.88, 95%CI = 1.52, 9.90), or neutral (OR = 1.54, 95%CI = 1.02, 2.30) feelings about vaccines in general, and disagreement with the statement “I am confident vaccines are safe” (OR = 2.30, 95%CI = 1.07, 4.93). Logistical difficulties including finding transportation (OR = 1.95, 95%CI = 1.01, 3.45), time (OR = 2.20, 95%CI = 1.18, 4.08), and need for two appointments within 30 days to get a second dose of a vaccine (OR = 2.64, 95%CI = 1.58, 4.44) were all associated with being unvaccinated. Unvaccinated respondents had higher odds of selecting “If my doctor recommended it” as a reason for getting a COVID-19 vaccine (OR = 1.44, 95%CI = 1.01, 2.04) (Table 2).

**Table 2.** Multivariable, adjusted binomial logistic regression models examining the association of demographics and attitudes and beliefs with not being vaccinated

Variable & reference group	Entire sample (*N* = 1,076)	
	OR (95% CI)	*p*
Age (ref: ≥75)^1^		
<50	7.68 (4.37–13.51)	**<0.001**
50–74	3.47 (2.16–5.57)	**<0.001**
Feelings about vaccines in general (1, “Very Negative” – 7, “Very Positive”; ref: Positive [6 or 7])		
Neutral (3, 4, or 5)	1.53 (1.02–2.30)	**0.039**
Negative (1 or 2)	3.88 (1.52–9.90)	**0.005**
“I am confident vaccines are safe.” (1, “Strongly disagree” – 5, “Strongly agree”; ref: Agree [4 or 5])		
Neutral (3)	1.37 (0.87–2.17)	0.176
Disagree (1 or 2)	2.30 (1.07–4.93)	**0.033**
Missing	0.33 (0.23–4.23)	0.396
“Which of the following are reasons that you would get a COVID-19 vaccine? (check all that apply)”		
It would be the best way to prevent me from getting COVID-19	0.39 (0.28–0.62)	**<0.001**
It is a way that I can contribute to ending the COVID-19 pandemic	0.55 (0.38–0.80)	**0.002**
If my doctor recommended it	1.44 (1.01–2.04)	**0.04**
“What do you think would be the biggest challenge for you getting a second dose of the vaccine after getting the first dose?”		
Transportation to healthcare appointment to get the vaccine	1.95 (1.01–3.45)	**0.022**
Finding a time to come in for the second visit	2.20 (1.18–4.08)	**0.012**
Scheduling two healthcare appointments within 30 d	2.64 (1.58–4.44)	**0.001**
Concerns that a second dose may increase the risk of side effects/adverse reactions	2.45 (1.63–3.67)	**<0.001**

## Discussion

This survey of 1,080 Veterans found that 65% of respondents were vaccinated. Unvaccinated respondents were younger, had greater concerns about COVID-19 vaccine safety/side effects, and negative attitudes regarding vaccines in general. Our results are consistent with previous findings from the Veteran^3^ and non-Veteran populations^3,9^ that younger age was associated with being unvaccinated against COVID-19. Unique to our study was the fact that logistical concerns around getting two vaccines were also associated with being unvaccinated. Potential solutions to this issue could include the expansion of programs designed to help Veterans with transportation to and from healthcare appointments including deploying community vaccination vans.

There are several strengths to this evaluation. We were able to survey Veterans from a geographically diverse section of the United States. Also, due to our sampling strategy, we were able to assess differences in race/ethnicity as well as sex and their association with vaccination. Our evaluation also had some limitations. We had lower response rates among those of racial and ethnic minorities which may have impacted our ability to detect vaccine intentions in those groups. Additionally, vaccinated individuals may have been more likely to respond than unvaccinated individuals.

This study identifies key attitudinal and belief factors associated with COVID-19 vaccination among Veterans. This prospect may have key implications for increasing uptake of the bivalent mRNA booster, which has only been received by 15.5% of the eligible population^10^. Finally, the role of practical barriers to receiving two-dose vaccines indicates that removing logistical challenges to vaccination will be key for facilitating uptake as the need for regular boosters or new vaccinations continues. Considering the critical role that vaccination will continue to play, not only for COVID-19 but for other communicable diseases, it is important to continue to make strides to improve the acceptance of vaccines.

## Supporting information

Wilson et al. supplementary material Wilson et al. supplementary material

## Supplementary material

For supplementary material accompanying this paper visit http://doi.org/10.1017/ash.2023.446. click here to view supplementary material
