# Supplementary material for: Age and beliefs about vaccines associated with COVID-19 vaccination among US Veterans
Source: Antimicrob Steward Healthc Epidemiol. 2023 Oct 23;3(1):e184. doi: 10.1017/ash.2023.446 (PMC10654943; doi:10.1017/ash.2023.446)
Supplement: Wilson et al. supplementary material [file S2732494X23004461sup001.docx]

**Table 2.** Beliefs and Attitudes Towards Vaccines and COVID-19 by Vaccination Status.

| Variable | Total | | | |
| --- | --- | --- | --- | --- |
|  | Unvaccinated (N=378) | Vaccinated (N=702) | p-value | |
| Perceived effectiveness of COVID-19 vaccines (1, “Not effective at all” – 7, “Very Effective”) |  |  | **<0.001** | |
| Ineffective (1 or 2) | 81 (21.4) | 16 (2.3) |  | |
| Neither effective nor  ineffective (3, 4, or 5) | 141 (37.3) | 137 (19.5) |  | |
| Effective (6 or 7) | 144 (38.1) | 535 (76.2) |  | |
| Missing | 12 (3.2) | 14 (2.0) |  | |
| Perceived Safety of COVID-19 Vaccines (1, “Very unsafe” – 5, “Very Safe”) |  |  | **<0.001** | |
| Unsafe (1 or 2) | 80 (21.2) | 6 (0.9) |  | |
| Neither safe nor  unsafe (3) | 105 (27.8) | 60 (8.5) |  | |
| Safe (4 or 5) | 190 (50.3) | 631 (89.9) |  | |
| Missing | 3 (0.7) | 5 (0.7) |  | |
| Extent worried about side effects from COVID-19 vaccines (1, “Not at all worried” – 7, “Very Worried”) |  |  | **<0.001** | |
| Not Worried (1 or 2) | 111 (29.4) | 442 (63.0) |  | |
| Somewhat Worried (3, 4, or 5) | 140 (37.0) | 175 (24.9) |  | |
| Worried (6 or 7) | 121 (32.0) | 79 (11.3) |  | |
| Missing | 6 (1.6) | 6 (0.8) |  | |
| Feelings about vaccines in general (1, “Very Negative” – 7, “Very Positive”) |  |  | **<0.001** | |
| Negative (1 or 2) | 45 (11.9) | 9 (1.3) |  | |
| Neutral (3, 4, or 5) | 125 (33.1) | 104 (14.9) |  | |
| Positive (6 or 7) | 202 (53.4) | 574 (82.2) |  | |
| Missing | 6 (1.6) | 11 (1.6) |  | |
| “I am confident vaccines are safe.” (1, “Strongly disagree” – 5, “Strongly agree”) |  |  | **<0.001** | |
| Disagree (1 or 2) | 55 (14.6) | 17 (2.4) |  | |
| Neutral (3) | 91 (24.1) | 69 (9.8) |  | |
| Agree (4 or 5) | 227 (60.0) | 603 (85.9) |  | |
| Missing | 5 (1.3) | 13 (1.9) |  | |
| Perceived impact of the COVID-19 pandemic on day-to-day life |  |  | **0.066** | |
| 1, “Not at all” | 76 (20.1) | 101 (14.4) |  | |
| 2, “A little” or 3, “A  moderate amount” | 86 (22.8) | 183 (26.1) |  | |
| 4, “A lot” | 88 (23.3) | 189 (26.9) |  | |
| 5, “A great deal” | 119 (31.5) | 204 (29.1) |  | |
| Missing | 9 (2.3) | 25 (3.5) |  | |
| Has anyone you care about been diagnosed with COVID-19? |  |  |  | |
| None | 115 (20.4) | 225 (32.1) | **0.583** | |
| Immediate Family | 142 (37.6) | 245 (34.9) | **0.384** | |
| Other Family | 112 (29.6) | 177 (25.2) | **0.118** | |
| Friends | 137 (36.2) | 250 (35.6) | **0.837** | |
| Work Colleagues | 73 (19.3) | 90 (12.8) | **0.004** | |
| “Which of the following are reasons that you would get a COVID-19 vaccine?” |  |  |  |  |
| It would be the best way to prevent me from getting COVID-19 | 201 (53.2) | 595 (84.8) | **<0.001** |  |
| It would be the best way to prevent others from getting COVID-19 | 182 (48.2) | 503 (71.7) | **<0.001** |  |
| So I would not have to worry about infecting other people | 181 (47.9) | 491 (69.9) | **<0.001** |  |
| I have a health condition that makes me more at risk from COVID-19 | 124 (32.8) | 319 (45.4) | **<0.001** |  |
| If my doctor recommended it | 102 (27.0) | 254 (36.2) | **0.002** |  |
| It is a way that I can contribute to ending the COVID-19 pandemic | 186 (49.2) | 542 (77.2) | **<0.001** |  |
| So life can go back to the way it was before the pandemic | 195 (51.6) | 506 (72.1) | **<0.001** |  |
| “What do you think would be the biggest challenge for you getting a second dose of the vaccine after getting the first dose?” |  |  |  |  |
| Transportation to health care appointments | 35 (9.3) | 36 (5.1) | **0.009** |  |
| Finding a time to come in for the second visit | 45 (11.9) | 24 (3.4) | **<0.001** |  |
| Scheduling two health care appointments within 30-days | 56 (14.8) | 35 (5.0) | **<0.001** |  |
| Remembering to show up for the second appointment | 53 (14.0) | 58 (8.3) | **0.003** |  |
| Concerns that a second dose may increase the risk of side effects | 104 (27.5) | 61 (8.7) | **<0.001** |  |
| I do not think a second dose of the vaccine will provide extra protection | 22 (5.8) | 10 (1.4) | **<0.001** |  |
